# Distinct role of the right temporoparietal junction in advantageous and disadvantageous inequity: A tDCS study

**DOI:** 10.3389/fnbeh.2022.1047593

**Published:** 2023-01-19

**Authors:** Shijing Wu, Shenggang Cai, Zhiqiang Dong, Hanqi Zhang

**Affiliations:** ^1^School of Economics and Management, South China Normal University, Guangzhou, China; ^2^Key Lab for Behavioral Economic Science and Technology, South China Normal University, Guangzhou, China

**Keywords:** inequity aversion, advantageous inequity, disadvantageous inequity, fairness, right temporoparietal junction, transcranial direct current stimulation

## Abstract

Fairness is a hallmark of humans' ability to maintain cooperative relationships with large numbers of unrelated others. It influences many aspects of daily life, from how people share their resources with partners to how policymakers shape income distribution policy. The right temporoparietal junction (rTPJ) is a hub of the mentalizing network and it has been proposed to play a key role in guiding human reciprocal behavior; however, its precise functional contribution to fair behavior in situations of advantageous and disadvantageous inequity remains unclear. The purpose of this study was to clarify the role of the rTPJ in relation to fair behavior in situations of advantageous and disadvantageous inequity by modulating the activation of the rTPJ through transcranial direct current stimulation (tDCS). Anodal tDCS at 1.5 mA over the primary visual cortex (VC) or rTPJ was performed and participants subsequently played a binary-choice version of the *Dictator Game*. We found that anodal tDCS over the rTPJ increased the participants' equity choices in the disadvantageous inequity situation but not in the advantageous inequity situation. The tDCS effect is moderated by sex and, in particular, the tDCS effect increases female equity choices. The results suggest that the rTPJ plays a distinct role in inequity aversion in these two types of inequity situations.

## 1. Introduction

A pervasive notion in social science is that human preferences and behaviors are sensitive to considerations of inequality (Fehr and Schmidt, 1999; Bolton and Ockenfels, 2000). People react negatively to receiving less than others (disadvantageous inequity aversion) or more than others (advantageous inequity aversion), and this is termed inequity aversion or a preference for fairness. This means individuals resist inequitable outcomes, that is, they are willing to give up some material payoff to move in the direction of more equitable outcomes (Bolton and Ockenfels, 2000). Several experiments conducted by Fehr et al. have confirmed this (Falk et al., 2003; Knoch et al., 2006; Fehr et al., 2008), which are similar to studies conducted by other researchers in the fields of psychology (Blake and McAuliffe, 2011; Güroglu et al., 2011) and neuroscience (Sanfey et al., 2003; Tricomi et al., 2010; Tricomi and Sullivan-Toole, 2015).

Different psychological mechanisms underpin the two types of inequity aversions. Disadvantageous inequity aversion has been found in many species (e.g., capuchins, macaques, chimpanzees, domestic dogs, and birds) (Brosnan and De Waal, 2003; Horowitz, 2012; Brosnan and de Waal, 2014; Shaw and Choshen-Hillel, 2017), whereas advantageous inequity aversion has only been seen in chimpanzees (Brosnan and de Waal, 2014). Moreover, human studies indicate that disadvantageous inequity aversion emerges earlier (Blake and McAuliffe, 2011) and is more pronounced than advantageous inequity aversion (Loewenstein et al., 1989; Brosnan and de Waal, 2014). Children as young as 3 years of age develop an aversion to disadvantageous inequity; they react negatively to receiving less than others and make costly rejections of disadvantageous unequal offers (Blake and McAuliffe, 2011; LoBue et al., 2011). However, it is only by approximately age 7 or 8 that children show robust evidence of advantageous inequity aversion and will sacrifice their own resources to avoid inequity between themselves and others (McAuliffe et al., 2017). Thus, we may infer that disadvantageous inequity aversion recruits emotion- and conflict-related cognitive processes, whereas advantageous inequity recruits relatively mature social and cognitive control processes (Gao et al., 2018).

The distinction between advantageous and disadvantageous inequity aversion is well supported by neural evidence (Morishima et al., 2012; Gao et al., 2018). Among many brain regions, the right temporoparietal junction (rTPJ) is known to play an essential role in making decisions based on inequity aversion in distribution games (Sanfey et al., 2003; Güroglu et al., 2011; Haruno et al., 2014). The previous study suggested the gray matter volume in the rTPJ is strongly associated with individuals' behavioral altruism in situations of advantageous inequity, but this was not found to be the case in situations involving aversion to disadvantageous inequity. The rTPJ, an area that has been shown to be implicated in perspective-taking tasks, is recruited when subjects face a trade-off between economic self-interest and other people's interests (Morishima et al., 2012).

Causal evidence for the role of the TPJ in advantageous inequity aversion is mixed. Soutschek et al. found that inhibitory transcranial magnetic stimulation (TMS) bursts of three stimuli at 50 Hz were repeated with a frequency of 5 Hz for 40 s over the rTPJ increased social discounting or the rate at which individuals become more selfish in more socially distant relationships (Soutschek et al., 2016). In contrast, one study found that participants who received right anodal/left cathodal tDCS over the TPJ were more averse to advantageous inequity (Luo et al., 2017), and putatively inhibitory repetitive TMS with triplets of pulses at 50 Hz, delivered in 5 Hz bursts for 40 s over the rTPJ, did not affect overall levels of advantageous inequity when participants selfishly held on to more resources for themselves (Brethel-Haurwitz et al., 2022). On the basis of such research, the precise functional contribution of the rTPJ to advantageous and disadvantageous inequity aversion remains unclear.

Sex has usually been taken into account as an important moderating variable related to social cognition and is included in the present study. A large body of evidence suggests that women are often more prosocial (e.g., generous, altruistic, and averse to inequality) than men, at least when other factors such as reputation and strategic considerations are excluded (Croson and Gneezy, 2009; Rand et al., 2016; Rand, 2017; Soutschek et al., 2017). Researchers have applied the *Dictator Game* and *Ultimatum Game* to investigate altruistic behaviors, with the results showing that female proposers give almost two times as much as male proposers to their paired recipient (Bolton and Katok, 1995; Eckel and Grossman, 1998; Croson and Gneezy, 2009), while female recipients are significantly more likely to accept lower offers than male recipients (Eckel and Grossman, 2001); the behavior of women is more sensitive to the social conditions of the experiment than the behavior of men.

Transcranial direct current stimulation is the application of a weak electrical current across a target brain region to modulate activity and establish a causal relationship between a behavior and a target brain region. tDCS modulates brain functions promoting or inhibiting neural activity in the target areas, and its molecular action has been hypothesized to involve both dopaminergic circuitries and neuroplasticity processes (Pettorruso et al., 2021). Compared with other brain stimulation techniques, tDCS is relatively cheaper, easy to use (Martinotti et al., 2019), with even fewer adverse effects (Brunoni et al., 2011; Aparício et al., 2016), painless, and noninvasive (Maréchal et al., 2017; Martin et al., 2017, 2019; Meyer et al., 2019; Wang and Zhang, 2021; Xiong et al., 2021; Zhang et al., 2022b). The mechanism of tDCS involves slight changes to plasticity *via* alteration of neurotransmitter activity (Stagg et al., 2018) or modulation of the spontaneous firing rate of the stimulated neurons and the membrane potentials (Stagg et al., 2018; Meyer et al., 2019). Anodal tDCS over target brain regions is thought to enhance neural excitability.

In the present study, we explored the causal roles of the rTPJ in aversion to advantageous and disadvantageous inequity. We administered anodal tDCS over the rTPJ (*n* = 37, 12 men) or primary visual cortex (VC, *n* = 34, 10 men) to exogenously enhance neural excitability. We used the VC group as a positive control group and employed a modified *Dictator Game* task to measure aversion to inequity. Our study empirically clarifies the neural mechanisms that regulate aversion to disadvantageous inequity situations.

## 2. Materials and methods

### 2.1. Participants

We recruited 71 healthy participants (mean age = 20.9 ±1.71 years, 49 women and 22 men) from South China Normal University. Participants received a monetary award for their time. All participants provided written informed consent in accordance with procedures approved by the South China Normal University Ethics Committee.

### 2.2. Experimental design

A positive-controlled, single-blind, mixed experimental design was applied. The between-group factor was the stimulation condition, and the participants were randomly assigned to one of two stimulation conditions: (1) anodal tDCS stimulation on the VC; (2) anodal tDCS stimulation on the rTPJ. The within-group factor was the inequity situation in which participants completed a modified dictator game task in both advantageous and disadvantageous inequity situations.

### 2.3. Transcranial direct current stimulation

We employed low-intensity tDCS to simply, painlessly, and noninvasively modulate brain activity (Xiong et al., 2019). A one-channel direct current stimulator (DC-stimulator; neuroConn, Ilmenau, Germany) and saline-soaked surface sponge electrodes (35 cm^2^) were used to administer the stimulation. Determined by the international electroencephalography 10–20 system (Homan et al., 1987), the VC and rTPJ were located at Oz and CP6 electrode sites, respectively. We chose the VC as a positive control to be consistent with noninvasive stimulation studies (Li et al., 2017). Those studies located the VC at the Oz; the VC is not involved in the advanced decision function and was usually used as a positive control. The reference electrode was placed on the contralateral arm of each participant.

The stimulation current faded in from 0 to 1.5 mA over 30 s, and a constant current of 1.5 mA was applied to the VC or rTPJ for 20 min after the current fade-in. The participants were instructed to wait for 20 min and do nothing during stimulation. After stimulation, participants completed the modified dictator game task.

### 2.4. Task and procedure

**Figure 1:**
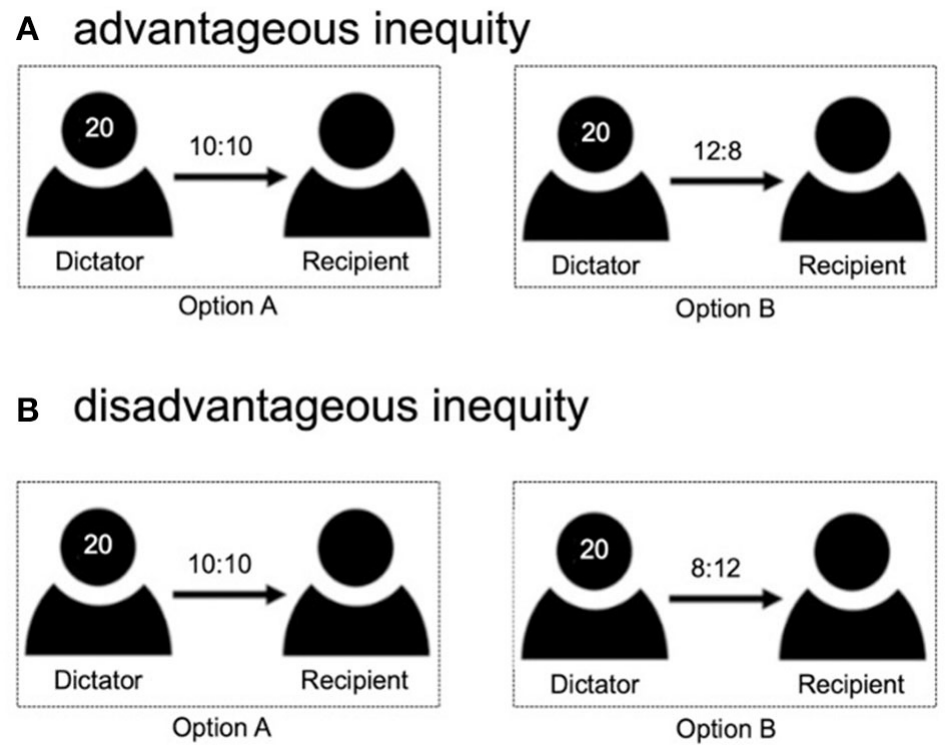
Illustration of different versions of the Dictator Game. The binary-choice version of the dictator game consisted of two options representing the payoffs that the participants (Dictator) and anonymous partners (Recipient) would earn. Option A was always an equal option, for example, “10 RMB for me, and 10 RMB for the other”; option B was an unequal option with different values, varied systematically across trials. **(A)** In the situations of advantageous inequity, participants (Dictator) always got more than their partner (Recipient) in the option B; **(B)** In the situations of disadvantageous inequity, participants (Dictator) always got less than their partner (Recipient) in the option B.

We used a binary-choice version of the dictator game to estimate the participants' aversion to inequity. The participants faced many decision problems and were instructed to choose one of two payoff options (option A or B) to allocate money (monetary unit: RMB yuan) to themselves and their anonymous partners. In the advantageous inequity situations, one option was an equal option where participants and their partner always got 10, and the other option was an unequal option where participants could get more than their partner (Figure 1A). In the disadvantageous inequity situations, in one option, participants and their partner always got 10, and in the other option, participants could get less than their partner (Figure 1B). The other option varied systematically across trials, in accordance with the inequity study by Gao et al. (2018).

The participants were randomly assigned to VC or rTPJ groups. After anodal stimulation, the participants were instructed to complete the modified dictator game task, with 77 randomly presented trials and the trial parameters were in line with a previous study (Gao et al., 2018). To dissociate self-payoff, other-payoff, and inequity, the specific values of self-payoff and other-payoff in the unequal options were determined by plotting several lines that passed through the 10/10 point and were evenly distributed in the self-other space (refer to Supplementary Table S1). Both the self-payoff and the other-payoff in the unequal options ranged from 2 to 31 RMB yuan. Meanwhile, the difference between the self-payoff and other-payoff of unequal options ranged from −0.1 to −24 RMB yuan in the disadvantageous inequity situation and 0.1 to 28 RMB yuan in the advantageous inequity situation (Zhang et al., 2022a). Two catch trials were selected; here, both options were equal but had different self-payoff and other-payoff values from the 10/10 options to exclude the participants who responded negatively. One trial was selected randomly and actualized after the experiment, determining the final payoffs for the participant and their partner.

## 3. Results

To directly characterize the treatment effects of the rTPJ on aversion to different situations of inequity, we employed a mixed-effect logistic regression using the lme4 package in R software. Participants' equity choices in each trial were the independent variable, in which the equity choice was denoted as 1, and the variable was otherwise coded as 0. Treatment, sex, and inequity were dependent variables. Treatment was a categorical variable with the VC as the baseline condition, while inequity was a categorical variable with advantageous inequity as the baseline condition.


$$\begin{array}{l} P\left(equity\right)=\beta_{0}+\beta_{1}\times Treatment+\beta_{2}\times Inequity+\beta_{3}\times Sex\\ \;+\beta_{4}\times Treatment\times Inequity+\beta_{5}\times Treatment\times Sex \end{array}$$

The results indicated that participants' equity choices were affected by the inequity situation (Table 1), with situations of disadvantageous inequity increasing participants' equity choices (β = 1.739, *p* < 0.001). The interaction between treatment and inequity was significant (β = 0.329, *p* = 0.015); in other words, anodal tDCS over the rTPJ increased the participants' equity choices in situations of disadvantageous inequity. We did not find sex effects (β = −0.072, *p* = 0.793) that have been previously identified in relation to social cognition and tDCS.

**Table 1:** Logistic regression coefficients indicating the effects of tDCS treatment, inequity situation, and sex on altruistic behavior.

	**Coefficient**	**SE**	***p*-value**	
Constant	−1.522	0.152	0.000	^***^
Treatment	−0.050	0.213	0.815	
Inequity (dis)	1.739	0.095	0.000	^***^
Sex	−0.072	0.260	0.783	
Treatment × Inequity	0.329	0.135	0.015	^*^
Treatment × Sex	−0.201	0.356	0.572	

^*^p < 0.05 and ^***^p < 0.001.

The model predicted the participant's probability [P (equity)] of making equity choices in each specific trial during the dictator game task based on tDCS treatment and sex in different inequity situations (Table 2). Treatment was a categorical variable with the VC as the baseline condition.

**Table 2:** Logistic regression coefficients indicating the effects of tDCS over rTPJ, and sex on equity choices in different inequity situations.

	**Advantageous inequity**	**Disadvantageous inequity**
	Coefficient (SE)	Coefficient (SE)
Constant	−2.086 (0.396)	0.202 (0.125)
Treatment	−0.482 (0.560)	0.392 (0.177)^*^
Sex	−0.265 (0.733)	−0.046 (0.230)
Treatment × Sex	0.795 (1.004)	−0.636 (0.315)^*^

* p < 0.05.

**Figure 2:**
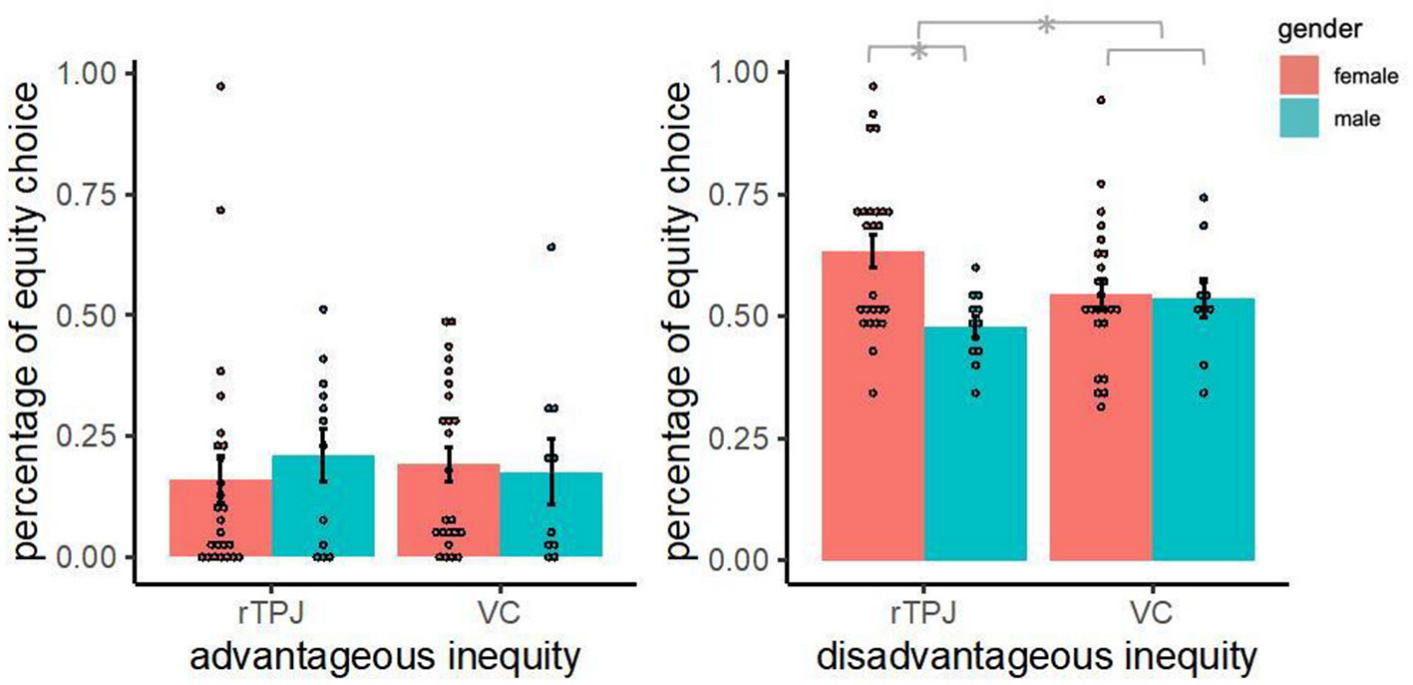
Percentage of equity choice. The bar represents the mean percentage of equity choices in each condition, and the error bar is standard error. ^*^p < 0.05.

In the disadvantageous inequity situations, we found that participants' selection of equity choices increased with treatment (β = 0.392, *p* = 0.027); tDCS over the rTPJ increased participants' aversion to disadvantageous inequity. The sex effect was not significant (β = −0.046, *p* = 0.842). However, the interaction between treatment and sex was significant (β = −0.636, *p* = 0.044). In the advantageous inequity situations, we found that the treatment effect (β = −0.482, *p* = 0.390), sex effect (β = −0.265, *p* = 0.718), and sex difference of tDCS effects (β = 0.795, *p* = 0.428) were not significant. As illustrated in Figure 2, the anodal tDCS over the rTPJ increased the participants' equity choices in the disadvantageous inequity situation but not in the advantageous inequity situation. The tDCS effect is moderated by sex, and in particular, the tDCS effect increases female equity choices.

## 4. Discussion

The findings showed that anodal tDCS over the rTPJ increased participants' equity choices in disadvantageous inequity situations and the effect was moderated by sex. However, the treatment effect, sex effect, and sex difference of tDCS effects were not significant in situations of advantageous inequity. These results may demonstrate that the rTPJ plays an important role related to fairness decisions in situations of disadvantageous but not advantageous inequity. The rTPJ may serve as a neural hub that signals the conflict between self-interest and moral considerations, which is consistent with the previous research (Obeso et al., 2018).

We found evidence that anodal tDCS over the rTPJ increased participants' equity choices in disadvantageous inequity situations. According to previous studies, the rTPJ is thought to contribute to several cognitive operations (Izuma, 2012; Schurz et al., 2014), and three different explanatory accounts have been put forward (Obeso et al., 2018). First, the rTPJ may be necessary for the motivation to do good to others (“other-regarding motivation”) (Hare et al., 2010; Jeurissen et al., 2014; Hutcherson et al., 2015; Park et al., 2017); second, the rTPJ represents the conflict between moral values and material concerns associated with sharing resources (“moral conflict”) (Berns et al., 2012; Morishima et al., 2012); third, the rTPJ manages individuals' social reputation by displaying socially desired behavior (“reputation”) (Izuma, 2012, 2013; Yomogida et al., 2017). Unlike advantageous inequity aversion, participants in disadvantageous inequity aversion have to weigh unfairness-evoked aversive responses against the conflicting personal financial benefit. Thus, disadvantageous inequity aversion recruits the processing of the conflict monitoring system (Gao et al., 2018). Increasing the neural excitability of the rTPJ might bias conflict between moral values and material benefits to moderate participants' equity choices in disadvantageous aversion.

The above results illustrate that the rTPJ plays a causal role in disadvantageous aversion, suggesting that the rTPJ may serve as a neural hub that signals the conflict between self-interest and moral considerations (Obeso et al., 2018). However, in advantageous inequity situations, we did not find that participants' choices of equity increased with treatment; tDCS over the rTPJ did not affect the aversion to advantageous inequity. This result is consistent with previous evidence showing that the rTPJ may play a role in differentiating between others when deciding how equitably to divide resources, but it may not play a general role in reducing selfishness by promoting an aversion to advantageous inequity (Brethel-Haurwitz et al., 2022). This finding indicates that the TPJ may not be critical for overall levels of aversion to advantageous inequity but instead may be critical for differentiating between social partners in resource allocation decisions (Brethel-Haurwitz et al., 2022). Moreover, behavioral studies have demonstrated that rejecting advantageous inequity requires more cognitive resources than those involved in rejecting disadvantageous inequity (van den Bos et al., 2006; Gao et al., 2018). Gao et al.'s study has provided neural evidence that the anterior insula (aINS), dorsolateral prefrontal cortex (DLPFC), and dorsomedial prefrontal cortex (DMPFC) underlie the processes of norm violation detection, cognitive control, and mentalizing in situations of advantageous inequity aversion (Gao et al., 2018). Thus, our findings showed that anodal tDCS over the rTPJ did not affect the aversion to advantageous inequity and may further demonstrate the differential neural mechanisms between advantageous and disadvantageous inequity.

We also found that the tDCS effect was moderated by sex. The interaction between treatment and sex was significant in disadvantageous aversion situations. During the inequity aversion situations, participants experienced a high-conflict situation and negatively perceived the presence of injustice. This effect was due to a social/emotional conflict that is more related to inequity aversion rather than being a cognitive issue (Vanutelli et al., 2020). However, men were more inclined to accept advantageous offers, while women were more inclined to accept disadvantageous offers (Vanutelli et al., 2020). This might be because inequity situations are more associated with emotion-related processing, especially for women, because accepting more than the opponent could result in prosocial reflections and unfairness avoidance (Friesdorf et al., 2015; Capraro and Sippel, 2017). This difference implies that women may need to expend more effort in paying attention to the varying financial costs/benefits and how these contrast with the constant moral value. Our results suggest that anodal tDCS on the rTPJ might affect the weighing of unfairness-evoked aversive responses against the conflicting personal financial benefit to moderate the equity choice in disadvantageous inequity aversion situations.

There exist limitations to the present study. First, we recruited a sufficient number of subjects and the population appears to be fairly homogeneous in terms of age and academic rank from the university. However, expanding this protocol to a wider population, including subjects of different ages, academic, and/or job statuses, could be more representative of the general population. We will consider this limitation in our future studies. Second, we applied a between-subject design rather than a within-subject design in our present study. The between-subject design is more conservative, whereas caution about carryover and demand effects should be taken when using the within-subject design. However, within-subject designs lend themselves to more powerful econometric techniques and, in many cases, are a closer match to a theoretical perspective (Charness et al., 2012; Zhang et al., 2022a). Although the between-subject design could eliminate potential learning of moral decisions (Obeso et al., 2018), the within-subject design can decrease the impact of inter-subject variability on the results. We will try to use a within-subject design to investigate the role of rTPJ in inequity aversion in the future.

## 5. Conclusion

Our data suggest that the rTPJ may serve as a neural hub that signals the conflict between self-interest and moral considerations. Furthermore, the rTPJ plays a distinct role in advantageous inequity aversion and disadvantageous inequity aversion. Anodal tDCS over the rTPJ affected equity choices by moderating the weighing of unfairness-evoked aversive responses against the conflicting personal financial benefit and sex in disadvantageous inequity situations. In future research, it will be worthwhile to apply the double dissociation paradigm to clarify whether altruism in these two inequity situations involves different mechanisms and whether these two situations operate independently of one another.

## Data availability statement

The raw data supporting the conclusions of this article will be made available by the authors, without undue reservation.

## Ethics statement

The studies involving human participants were reviewed and approved by the South China Normal University Ethics Committee. The patients/participants provided their written informed consent to participate in this study.

## Author contributions

SW, SC, ZD, and HZ participated in the design of this study, carried out the study, constructed the overall framework of the study, and modified and polished it. SW and HZ performed the statistical analysis, collected important background information, and drafted the manuscript. All authors read and approved the final manuscript.
