# Supplementary material for: Distinct role of the right temporoparietal junction in advantageous and disadvantageous inequity: A tDCS study
Source: Front Behav Neurosci. 2023 Jan 19;16:1047593. doi: 10.3389/fnbeh.2022.1047593 (PMC9892459; doi:10.3389/fnbeh.2022.1047593)
Supplement: Supplementary file 1 [file Table_1.DOCX]

**Table S1. Unequal option settings for the Experiment** (Gao et al. 2018)

| Self | Other | Number of trials | Self vs. Other | Inequity level | Inequity type |
| --- | --- | --- | --- | --- | --- |
| 4 | 28 | 2 | -24 | High | Disadvantageous |
| 10 | 30 | 2 | -20 | High | Disadvantageous |
| 6 | 22 | 2 | -16 | High | Disadvantageous |
| 2 | 18 | 2 | -16 | High | Disadvantageous |
| 10 | 26 | 1 | -16 | High | Disadvantageous |
| 15 | 30 | 2 | -15 | High | Disadvantageous |
| 4 | 16 | 2 | -12 | High | Disadvantageous |
| 1 | 13 | 2 | -12 | High | Disadvantageous |
| 14 | 26 | 1 | -12 | High | Disadvantageous |
| 10 | 22 | 2 | -12 | High | Disadvantageous |
| 12 | 22 | 2 | -10 | Low | Disadvantageous |
| 20 | 30 | 1 | -10 | Low | Disadvantageous |
| 8 | 16 | 2 | -8 | Low | Disadvantageous |
| 4 | 12 | 2 | -8 | Low | Disadvantageous |
| 10 | 18 | 2 | -8 | Low | Disadvantageous |
| 7 | 13 | 2 | -6 | Low | Disadvantageous |
| 12 | 18 | 2 | -6 | Low | Disadvantageous |
| 25 | 30 | 1 | -5 | Low | Disadvantageous |
| 7 | 11 | 2 | -4 | Low | Disadvantageous |
| 29.9 | 30 | 1 | -0.1 | Low | Disadvantageous |
| 2 | 2 | 1 | 0 | - | Equal (Catch trial) |
| 18 | 18 | 1 | 0 | - | Equal (Catch trial) |
| 30 | 29.9 | 1 | 0.1 | Low | Advantageous |
| 11 | 8 | 2 | 3 | Low | Advantageous |
| 12 | 8 | 2 | 4 | Low | Advantageous |
| 11 | 6 | 2 | 5 | Low | Advantageous |
| 30 | 25 | 1 | 5 | Low | Advantageous |
| 18 | 12 | 2 | 6 | Low | Advantageous |
| 14 | 6 | 2 | 8 | Low | Advantageous |
| 16 | 8 | 2 | 8 | Low | Advantageous |
| 18 | 10 | 2 | 8 | Low | Advantageous |
| 12 | 3 | 2 | 9 | Low | Advantageous |
| 22 | 12 | 2 | 10 | High | Advantageous |
| 30 | 20 | 1 | 10 | High | Advantageous |
| 22 | 10 | 2 | 12 | High | Advantageous |
| 26 | 14 | 1 | 12 | High | Advantageous |
| 17 | 3 | 2 | 14 | High | Advantageous |
| 30 | 15 | 2 | 15 | High | Advantageous |
| 22 | 6 | 2 | 16 | High | Advantageous |
| 26 | 10 | 1 | 16 | High | Advantageous |
| 30 | 10 | 2 | 20 | High | Advantageous |
| 31 | 3 | 2 | 28 | High | Advantageous |

**References**

Gao, X., Yu, H., Sáez, I., Blue, P. R., Zhu, L., Hsu, M., & Zhou, X. (2018). Distinguishing neural correlates of context-dependent advantageous- and disadvantageous-inequity aversion. *Proceedings of the National Academy of Sciences*, *115*(33), E7680–E7689. https://doi.org/10.1073/pnas.1802523115
